# Secure Localization in the Presence of Colluders in WSNs

**DOI:** 10.3390/s17081892

**Published:** 2017-08-17

**Authors:** Wei Shi, Michel Barbeau, Jean-Pierre Corriveau, Joaquin Garcia-Alfaro, Meng Yao

**Affiliations:** 1 School of Information Technology, Faculty of Engineering and Design, Carleton University, Ottawa, ON K1S 5B6, Canada; 2 School of Computer Science, Carleton University, Ottawa, ON K1S 5B6, Canada; barbeau@scs.carleton.ca (M.B.); jeanpier@scs.carleton.ca (J.-P.C.); 3 Institut Mines-Telecom, Paris-Saclay University, Telecom SudParis, CNRS Samovar UMR 5157, 9 Rue Charles Fourier, 91000 Evry, France; joaquin.garcia_alfaro@telecom-sudparis.eu; 4 Nokia Networks, 600 March Road, Ottawa, ON K2K 2T6, Canada; meng.yao@nokia.com

**Keywords:** colluders, detection, secure localization, wireless sensor network

## Abstract

We address the challenge of correctly estimating the position of wireless sensor network (WSN) nodes in the presence of malicious adversaries. We consider adversarial situations during the execution of node localization under three classes of colluding adversaries. We describe a decentralized algorithm that aims at determining the position of nodes in the presence of such colluders. Colluders are assumed to either forge or manipulate the information they exchange with the other nodes of the WSN. This algorithm allows location-unknown nodes to successfully detect adversaries within their communication range. Numeric simulation is reported to validate the approach. Results show the validity of the proposal, both in terms of localization and adversary detection.

## 1. Introduction

In a wireless sensor networks (WSNs), several small devices (hereinafter referred to as sensor nodes) collaboratively exchange information. Most existing WSN applications include environmental monitoring [1] and tracking of goods and people [2]. WSNs usually work in a hostile environment. Therefore, they are vulnerable to various malicious attacks such as cyber attacks [3] and wormhole attacks [4]. In several WSN applications, the ability to localize a sensor plays a critical role [4,5,6,7,8,9,10,11].

Most of these applications are complemented by geographic routing and location-based authentication which, in turn, require node position determination techniques. If such a process does not include security measures, the WSN will eventually suffer from location-based threats conducted by adversarial scenarios, including both Sybil and sinkholing attacks [12,13]. Attacks to the localization process may successfully interfere with the objectives of a given WSN application. Consider, for instance, a WSN-based system that monitors forest fires. Localization errors can incorrectly report in which precise geographic area to intervene. Similarly, erroneous positioning in critical infrastructure scenarios can report incorrect information to security and safety operators. It is thus crucial to use a secure localization algorithm, in order to guarantee the accuracy of the reported position of the nodes of a WSN.

Localization involves two steps: first, acquiring data, and then conducting position calculation. During either of these two steps, an (internal or external) [14] attack can disrupt the localization process by having a node either sending false information or by simply replaying data previously obtained under normal operations. Either case may disrupt the system, since the network may end up with nodes advertising geographical positions that have been wrongly computed. Security measures have been created to mitigate the impact of these attacks. However, issues pertaining to collusion remain: (1) How do colluders work together? and (2) To which extent can they interfere with the localization process?

Collusion may take many forms in a WSN. Collaborating attackers could announce false information in a coordinated manner and/or position themselves into evasion patterns (for example to disclose collinear positions that fool detection). Both cases may result in calculating an incorrect position for a node. Additionally, such attackers could target other data such as the reputation lists used in several locational algorithms [15,16,17,18]. Such a possibility is downplayed in current approaches to localization. Indeed, it is often assumed that (a) such reputation lists are incorruptible and that (b) coordination of adversarial nodes does not affect the localization process. Some existing studies do consider collusion, that is, they assume that a group of malicious sensor nodes does collaborate to alter the information they exchange with (i.e., receive from and forward to) other nodes. They number few and are limited. Let us elaborate.

First consider the situation where the adversaries evade detection by positioning themselves at ‘blind’ positions of the detection algorithm [16]. For example, an evil ring attacker *era* (*ibid.*) fools location-unknown nodes that rely on trilateration to compute their geographic location using *era*’s position. Such an attack is not detectable by algorithms that detect *simple liars*, where a ‘simple liar’ is taken to be a sensor that sends out randomly generated fake coordinates to its neighbours. We have presented elsewhere [16,19] an algorithm able to detect and handle a single evil ring attacker. We emphasize that this algorithm cannot handle localization in the presence of multiple evil ring attackers. We also remark that the few techniques that exist in the literature to handle colluders (hereafter also referred to as colluding attackers or colluding sensors) cannot handle the evil ring attack.

Next, let us understand other limitations of existing algorithms for handling colluders. Typically, these algorithms assume the existence of trusted third parties (e.g., a trusted base station, such as in [20]). This is problematic for several reasons. First, the trusted third parties must periodically broadcast positioning validation data to all the system nodes. Second, the only element of the system that can detect colluders is, in fact, the trusted third party. Third, these solutions require secure communication to the third party, which does not allow for decentralized processing (though this is an key requirement for WSNs). In turn, this imposes an important overhead and complexity on the final system. Also, heavy traffic to and from the base station is likely to compromise the lifespan of neighbour nodes. Ultimately, relying on a trusted third party (which, in effect, disables true distributed processing) and failing to handle attacks such as the evil ring one, completely jeopardizes such solutions. Instead, what is required is a distributed algorithm enabling the detection of colluders (including evil ring attackers) by the nodes of a WSN in a totally decentralized way [7,21].

In this paper, we present such an algorithm, which proceeds from our previous work on the evil ring attack. Most importantly, like other studies addressing localization in the presence of colluders, such colluders are allowed to alter not only coordinates but also other messages used for localization [22]. In our approach, any location-unknown node is able to identify in a decentralized way colluders within its communication range.

The rest of the paper is organized as follows. A review of the literature is conducted in Section 2, with a brief overview of our work on the evil ring attack and how it is different from the algorithm we present here. Section 3 provides the background, model and assumptions of the detection algorithm originally published in [19] for the case of a single evil ring attacker [19]. It also introduces a colluding attack model that includes three categories of colluding attackers. Section 4 presents our solution for detecting the presence of any of these three categories of colluding attackers and Section 5 provides a detailed correctness analysis of the proposed algorithm. In Section 6 we discuss our comprehensive set of simulation results. Section 7 concludes the paper.

## 2. Related Work

The impact of the wormhole attack on the DV-Hop localization algorithm is analysed in [4]. Based on the analysis results, a label-based DV-Hop secure localization scheme is proposed to defend against the wormhole attack. The proposed scheme assumes an ideal and homogeneous communication range for all the sensors. The simulation results prove the effectiveness of the proposed scheme.

A secure and scalable geographic opportunistic routing (SGOR) protocol [7] is proposed to defend against a wide range of attacks. SGOR employs a distributed location verification algorithm to address the location-spoofing attack. In order to detect this attack, the sensor nodes exchange messages between them and records the received signal strength (RSS) measurements. In the routing layer, an indirect trust model is proposed to response to different attacks based on the location verification algorithm. Simulation results prove that SGOR has an excellent performance with acceptable overhead under various attacks.

Another secure region-based geographic routing (SRBGR) protocol is proposed in [23]. The basic idea behind SRBGR is to increase the number of legitimate nodes participating in communication process when the number of attackers increases by applying different message contention priorities. SRBGR uses a bound dynamic window to provide sufficient collection time for packets. It also uses the verification cost to validate node’s location in order to identify and isolate the attacker during priority selection criteria. Simulation results demonstrate that SRBGR increases the network performance in terms of the packet delivery ratio and isolates attacks such as Sybil and blackhole attacks.

A secure and reliable prediction-based target tracking (SRPTT) protocol [6] is proposed to consider both security and object tracking tasks simultaneously. The basic idea behind SRPTT is to ensure security using reputation based trust concept for individual sensor nodes. The possible attack scenarios for SRPTT are analysed and simulation results show that the proposed protocol allows the network to retain the reliability of tracking data even in the presence of compromised nodes, thereby achieving secure and reliable object tracking process.

The work in [24] analyses existing techniques for collusion prevention. Results show their inadequacy for prevention of colluders in WSN scenarios. The work in [25] addresses bounded errors conditions to guarantee the security of the process conducted by sensor nodes using a two-dimensional distance-based estimation process, executed in the presence of cheating beacon nodes. Three algorithms driven by distance-based techniques are developed to guarantee bounded error localization under cheating beacons. Cheating beacons are defined as beacons providing a false distance between nodes. The goal of the attackers generating the cheating beacons is to lead location-unknown nodes to miscalculate their position. Accuracy and efficiency of the algorithms are measured via numeric simulation.

A positioning process called tolerant majority colluding attacks (TMCA) [18] assumes the cooperation of non-beacon WSN neighbour nodes in order to address adversarial scenarios such as wormholes, replay and Sybil attacks. TMCA is validated under situations where the number of malicious nodes is larger than the number of benign nodes. TMCA allows location-unknown nodes to compute their position using distance bounding techniques. The underlying technique relies on the use of *maximum likelihood estimation*, in order to correlate the coordinates and the distance measurements received from a reference beacon. Authors in [24] observed a lack of evidence with regard to collaboration (e.g., amount of exchanges) or collusion of adversarial nodes in TMCA.

The authors in [24] also illustrate attack possibilities in some other protocols similar to TMCA reported in [26]. The following observations are made. First, provers must properly compute their distance with regard to honest verifiers, including those situations in which adversaries exist. Second, provers must compute upper bounds on their distance to dishonest verifiers, also including situations where colluders do exist. If the aforementioned premises hold, then provers may mutually authenticate verifiers by using cryptographic solutions (global synchronization of messages is required).

Positioning algorithms in [27,28,29] enable location-unknown nodes to geolocate their coordinates in a WSN despite the presence of malicious anchor nodes lying about their position in the network. A first algorithm, referred to as *majority-three neighbour signals*, uses trilateration to calculate the position of location-unknown nodes [30]. The algorithm relies on the use of majority decision rules to obtain the positions. Triplets determining a location in disagreement with the majority are considered to contain malicious anchor nodes. In [16], an attack referred to as *evil ring* is presented against the *majority-three neighbour signals* algorithm. Attackers are assumed to provide wrong positions, while remaining undetectable by the *majority-three neighbour signals* since the victims of the attack derive consistent positions with the majority. When asked, the malicious nodes report fake positions that sit on a circle centred at the victim’s location and with a radius corresponding to the distance separating attackers and victims. The calculation of the distance between attackers and victims remains consistent. Thus, location-unknown sensor nodes correctly determine their location but fail at properly identifying the position of that malicious node. This is because an evil ring attacker is a ‘smart liar’ that does not merely send out randomly generated fake coordinates to its neighbours (which is what a simple liar does). Instead, an evil ring attacker computes a special location that will further interfere with the localization computation of other sensors. Such behaviour is undetectable by any sensor running any of the previous detection algorithms that use trilateration to determine sensor position and detect liars.

The algorithm presented in [16] handles the localization issue by *cross-checking* the information provided by an evil ring attacker. More precisely, the solution relies on requesting the locations of all neighbouring nodes and computing every possible three-neighbour combination using majority decision rules. Then, a list is created to store all those triplets that agree with the majority. The computed position and the cross-check lists are broadcasted to neighbours, which allows for the detection of the evil ring attacker (i.e., the smart liar). Sensor nodes wait until they receive the equivalent lists from two other neighbours and compare the results. Every neighbouring node that is consistently reported with the same position in the three lists is reported as truth-teller—or as a liar otherwise.

The proposals presented in [16,27,28,29] assume collusion-free dense networks. The absence of collusion in TMCA and in the *evil ring* attack is a typical assumption. As previously mentioned, very few studies pertaining to secure localization have investigated attacks perpetrated by colluders.

Let us consider models that do address collusion, for example, the solution presented in [31]. This solution assumes location information that can be received and forwarded by collaborating malicious nodes. However, as mentioned in the previous section, this solution fails to detect an evil ring attack and has other limitations.

## 3. Background, Models and Assumptions

Let K be the complete set of location-known sensor nodes in a WSN. These nodes know their position in the system beforehand. They may be an *anchor* node (e.g., nodes that were manually configured by operators of the system) or may use a GPS for self-positioning [32,33] or may have their location computed a priori. Conversely, let U be the set of location-unknown sensor nodes that will try to determine their own position in the system by collaborating with other nodes within their communication range. We assume that all the sensor nodes are deployed on a two-dimensional plane. Sensor nodes K are assumed to be either truth-tellers (i.e., never intentionally provide false information to neighbours); or *liars* [16] that can intentionally lie about their location in the system.

In the presence of liars, exact boundaries about the exact number of location-known neighbours required to correctly conduct the location process exist [16]. For instance, results reported in [27,28,29] provide different boundaries with regard to the exact adversary models and robustness of the process. The higher the robustness, the lower number of liars that might be handled by the localization process. The following boundary provides an example under worst case adversaries and highest robustness level:

(1) |N|3>2ℓ3+|N|-ℓ1·ℓ2+|N|-ℓ2·ℓ1

where |N| is the number of neighbouring nodes of a location-unknown node conducting the process, out of which exactly *ℓ* nodes are liars. According to the previous function, seven nodes are required in case the number of liars is exactly one (cf. [29] for further details); 11 nodes in the case of two liars; 16 nodes in case of three liars; and so on.

Now, in order to address collusion, let C⊂K denote a set of *colluders* that jointly perpetrate an attack. A location-known node $C_{i}\in C$ is not only a *liar* (i.e., inasmuch as it systematically reports a fake location to other sensor nodes in the network) but can also send out other false information, such as fake lists about its neighbouring nodes. We emphasize, once more, that the presence of such colluders in a WSN invalidates existing solutions for secure localization.

**Figure 1:**
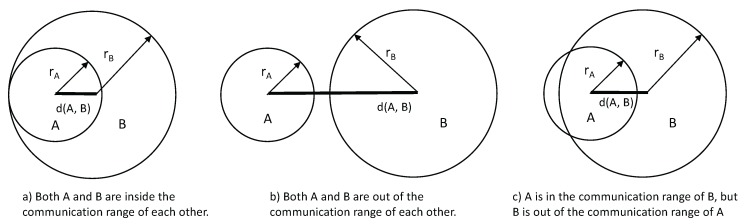
Communication range scenarios of two sensors nodes *A* and *B*. (**a**) Both *A* and *B* are inside the communication range of each other. (**b**) Both *A* and *B* are out of the communication range of each other. (**c**) *A* is in the communication range of *B*, but *B* is out of the communication range of *A*.

**Definition** **1.** *Sensors A and B have bi-directional communication if both A is in B’s communication range and A is in B’s communication range, as illustrated in Figure 1a. They have no communication if neither A nor B is in the communication range of the other, as illustrated in Figure 1b. They have single direction communication if either only A is in B’s communication range or vice versa, as illustrated in Figure 1c.*

We propose an algorithm to allow a sensor *A* to obtain its own position in the system and detect colluders that are within its bi-directional communication range despite the presence of these colluders. Such detection requires understanding the nature of threats (jeopardizing the accuracy of the localization process) originating from colluders. Clearly, reducing communications between sensor nodes limits the likelihood of such threats succeeding. Consequently, in our proposed algorithm, the only messages we allow between sensor nodes consist in their coordinates and a series of verification lists (hereafter denotes as *cross-check lists* or *CCL*s for short). We do assume periodic exchanges between neighbours (e.g., to exchange routing data) are secured and cannot be attacked.

Every node is identified by its coordinates. Authentication (via identity theft) and data integrity may be compromised in a colluding attack. It is important to note that our proposed solution does not rely on any form of authentication nor cryptography.

Our solution identifies three categories of colluders. All attacks we consider depend on a corrupted *CCL* being sent. Which category an attacker belongs to depends on how it reports its location. A category one attacker lies by supplying fake locations generated at random. A category two attacker provides fake location in cooperation with two other malicious nodes, without aiming at escaping detection. For instance, we include in this category nodes that may provide false locations in order to affect the positioning process of those location-unknown sensor nodes requesting collaboration from their neighbours Such colluders do not implement any attack techniques to evade detection from neighbouring nodes. Finally, category three attackers consistently lie in cooperation with other colluders, with the goal of misleading the positioning process of location-unknown nodes, as well as enforcing techniques to evade detection. Category three attackers are assumed to conduct *evil ring* attack techniques (reported in [16]), for example by reporting false positions on a circle centred at the victim’s location and of a radius corresponding to the distance between the attacker and victim. Finally, we emphasize that colluders of all three categories can compromise data integrity, for example by forging colluded *CCL*s.

Let $r_{A}$ denote the communication range of a location-unknown sensor *A*. Let $d_{A}$ be a disc of radius $r_{A}$ centered at *A*. Let K∈K be a location-known sensor with communication range $r_{K}$. Let $d_{K}$ be a disc of radius $r_{K}$ centred at *K*. Let *l* denote the number of truth-telling sensors that: (1) are in the intersection of $d_{A}$ and $d_{K}$; and (2) have *bi-directional* communication with both *A* and *K*. Let *m* denote the exact amount of colluders that intersect $d_{A}$ and $d_{K}$. We postulate that for *A* to systematically detect all colluders, the number of truth-telling sensors *l* should be at least two more than *m*. In what follows, we present algorithmic solutions to address the third category of colluders, which are not detectable by previous algorithms in the literature. We emphasize that algorithms that can handle colluders of category three clearly can also handle the two other categories of colluders.

## 4. Super Cross-Check (SCC) Algorithm

**Figure 2:**
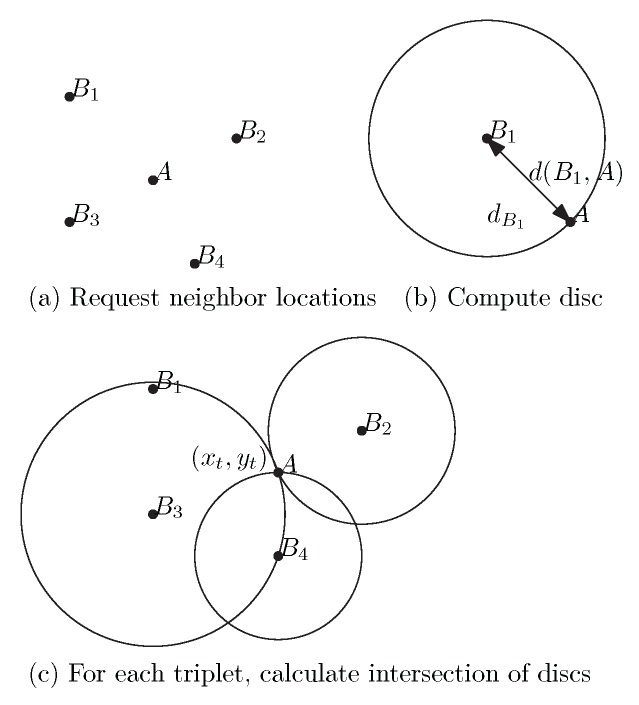
Detailed example: (**a**) In this example, sensor *A* has four neighbours, i.e., K contains $B_{1},B_{2},B_{3}$ and $B_{4}$. For i=1,2,3,4, sensor *A* (1) requests and gets the location $\left(x_{i},y_{i}\right)$ of every neighbour $B_{i}$; and (2) derives each self-to-neighbour $B_{i}$ distance. (**b**) Using the location of every neighbour $B_{i}$ and distance ($d(B_{i},A)$), a disc is calculated ($d_{B_{i}}$). Here this is shown for neighbour $B_{1}$. (**c**) For each possible triplet *t* over the set of discs obtained in (**b**), calculate the intersection point $\left(x_{t},y_{t}\right)$ of this triplet of discs. This is shown here for *t* equal to $\left\{B_{2},B_{3},B_{4}\right\}$. In this example, there are four possible combinations of three, that is, four triplets. Assuming here that all neighbours are truth-tellers and mutually within communication range, they will be all in agreement (up to this point: Lines 1 to 4 of Algorithm 1). The CCL of *A* contains $B_{1},B_{2},B_{3}$ and $B_{4}$, and their coordinates (Lines 6 to 8 of Algorithm 1). The CCLs obtained from neighbours are all consistent (Lines 9 to 11 of Algorithm 1). Each neighbour is consistently listed in three CCLs of neighbours. For example, $B_{1}$ is listed in the CCLs of $B_{2},B_{3}$ and $B_{4}$, with the same coordinates. Each neighbour gets a score of 3 (Lines 12 to 18 of Algorithm 1). Consequently, each neighbour is labelled truth-teller (Lines 19 to 25 of Algorithm 1).

Algorithm 1 outlines the steps of *super cross-check* (SCC). It assumes a sensor node *A* that executes two main processes. First, it tries to compute a consistent position in the system by requesting the position of every location-known sensor node in its neighbourhood Then, it labels its neighbours as either truth-tellers or colluders. The main steps to identify the colluders are the following. Once node *A* computes its position, it creates a cross-check list (*CCL*) for every neighbour in K. Then, node *A* exchanges its *CCL* with those sensor nodes in its communication range, in order to identify colluders. Upon request, every neighbour of *A* sends out its *CCL*. Only colluders would send *CCL*s with wrong information (with respect to the truthful information provided by the truth-tellers). For example, a colluder may purposely include false positions about nodes that should have been excluded from its *CCL*. If enough colluders collectively report exactly the same information, then node *A* will be deceived. The attack can be detected by using a traditional voting technique. Upon receiving a *CCL* from each neighbour, *A* computes for every neighbour either positive and negative scores. If a neighbour received two more positive scores than negative scores, then *A* concludes that such a node is a truth-teller. Otherwise, the node would be labelled as a colluder. In Figure 2 we provide a detailed example illustrating how algorithm SCC works.


**Algorithm 1** Super cross-check (SCC)
1:**repeat**2:    *// sensor A requests the location of all its neighbours and computes the point of*3:    *// intersection $\left(x_{t},y_{t}\right)$ for each triplet t of nodes $B_{i},B_{j},B_{k}$ in its neighbourhood;*4:**until** a consistent position is derived from the majority of triplets;5:Once the above process returns a consistent position6:*// sensor A accepts $\left(x_{t},y_{t}\right)$ as its position in the system, adds every triplet*7:*// ($B_{i}$; $B_{j}$; $B_{k}$) in agreement with $\left(x_{t},y_{t}\right)$ to its cross-check list (CCL), and*8:*// broadcasts both its location $\left(x_{t},y_{t}\right)$ and its CCL to all its neighbours;*9:**repeat**10:    *// sensor A requests the CCL of each neighbour and includes the information in a set K;*11:**until** all the neighbours of *A* are processed12:**for** each node in *K* whose communication circle intersects with *A*
**do**13:    **if** the node is reported in both the CCL of *A* and the CCL of *A*’s neighbours **then**14:        *// the node gets a positive score;*15:    **else**16:        *// the node gets a negative score;*17:    **end if**18:**end for**19:**for** each node in *K* whose communication circle intersects with *A*
**do**20:    **if** the node has twice positive scores than negative scores **then**21:        *// the node is labelled as a truth-telling node*22:    **else**23:        *// the node is labelled as a colluder*24:    **end if**25:**end for**

## 5. Correctness Analysis

We demonstrate that a colluder is uncovered by Algorithm 1, despite its insertion in the *CCL* of *A*.

**Lemma** **1.** *A location-unknown sensor A executing Algorithm 1 detects all in-range colluders.*

**Figure 3:**
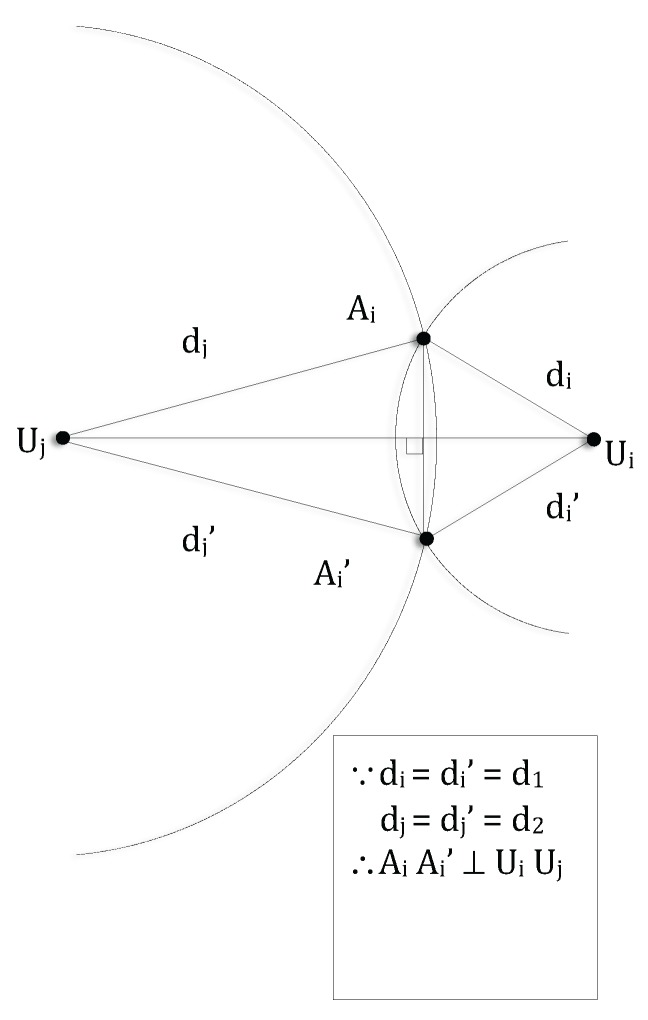
Let $A_{i}$ be an evil-ring attacker and $A_{i}^{\prime }$ be its fake location. Any sensor on the line that is perpendicular to $A_{i}A_{i}^{\prime }$ and passing through $A_{i}A_{i}^{\prime }$’s midpoint cannot detect $A_{i}$ as an evil-ring attacker. Given no three sensors are collinear (by assumption), there is no other location-unknown sensor other than $U_{i}$ and $U_{j}$ that can be fooled by $A_{i}$.

**Proof.**  Let $A_{i}$ be a colluder. According to Lines 1–11 in Algorithm 1, the false coordinates of $A_{i}$ are listed in the *CCL* of *A*. In the intersection of the discs $d_{A_{i}}$ and $d_{A}$, let *l* be the number of truth-telling sensors and *m* be the number of attackers colluding with $A_{i}$. We assume that l≥m+2 and that each of these *l* truth-telling sensor node has *bi-directional* communication with *A*. In the worst case, the false coordinates of $A_{i}$ are present in the *CCL* of *m* colluders. It is proved in [16] Lemma 2 that $A_{i}$ can fool a maximum of two truth-telling sensors (e.g., $U_{i}$ and $U_{j}$ in Figure 3). This means that there is potentially another truth-telling sensor node attacked by $A_{i}$. If this happens, then node $A_{i}$ is included in the *CCL* of that truth-telling sensor node. Hence, *A* receives from its neighbours a maximum of m+1 *CCL*s including $A_{i}$. According to Lines 12–18 in Algorithm 1, *A* gives a positive credit at most m+1 times and a negative credit at most l-1 times to $A_{i}$. Since i≥m+2, we have that l-1≥m+1. Therefore, more negative credits than positive credits are attributed to $A_{i}$. According to Lines 19–25 in Algorithm 1, $A_{i}$ is declared a colluder by *A*. ☐

**Lemma** **2.** *A location-unknown sensor A executing Algorithm 1 detects all in-range truth-telling sensor nodes, when the number of truth-telling sensor nodes i is at least two more units greater than j, the number of colluders.*

**Proof.**  If *B* is a truth-telling sensor node, it should be present in the *CCL*s of *l* truth-telling sensor nodes in the intersection of the discs $d_{A}$ and $d_{B}$. In the worst case, all *m* colluders remove the coordinates of *B* from their *CCL*s. According to lines 12–18 in Algorithm 1, *A* assigns *l* positive credits and *m* negative credits to *B*. Given the number of truth-telling sensors *l* is at least two more units greater than *m*, namely, l≥m+2, and according to lines 19–25 in Algorithm 1, *A* will declare *B* as a truth-telling sensor. ☐

From Lemmas 1 and 2, we derive the following theorem:

**Theorem** **1.** *A location-unknown sensor A executing Algorithm 1 detects all in-range colluders and truth-telling node sensors.*

## 6. Experimental Results

We present in this section some numeric simulation results. We implemented our algorithms and scenarios under the OMNeT++ framework [34]. Two different set of simulations are reported hereinafter. First, we assume scenarios where the sensor nodes are uniformly deployed. Second, we assume a random deployment of nodes. We assume that a neighbourhood of over 30 active nodes creates a major interference condition and makes communications ineffective. The performance of the SCC algorithm is evaluated as follows. We measure and compare the successful detection colluders with regard to the performance and cost of the *cross-check* (CC) algorithm presented in [16]. The CC algorithm is reproduced in our work as Algorithm 2, for the convenience of readers (see [16] and citations thereof, for the correctness and complexity analysis of Algorithm 2).

**Algorithm 2** Cross-Check (CC)1:**repeat**2:    *// sensor A requests the location of all its neighbours and computes the point of*3:    *// intersection $\left(x_{t},y_{t}\right)$ for each triple t of nodes $B_{i},B_{j},B_{k}$ in its neighbourhood;*4:**until** a consistent position is derived from the majority of triplets;5:*// sensor A accepts $\left(x_{t},y_{t}\right)$ as its position in the system, adds every triplet*6:*// ($B_{i}$; $B_{j}$; $B_{k}$) in agreement with $\left(x_{t},y_{t}\right)$ to its cross-check list (CCL), and*7:*// broadcasts both its location $\left(x_{t},y_{t}\right)$ and its CCL to all its neighbours;*8:**repeat**9:    *// sensor A requests the CCL of its neighbours;*10:**until** consistency of *A*’s CCL and two CCLs from two different neighbours11:**for** each node $B_{i}$ in *A*’s neighbourhood **do**12:    **if** the position of $B_{i}$ is consistent in all three *CCL*s **then**13:        $B_{i}$ is labelled as a truth-teller and is added to the neighbourhood list of *A*;14:    **else**15:        $B_{i}$ is labelled as a colluder and is excluded from *A*’s neighbourhood list;16:    **end if**17:**end for**

### 6.1. First Set of Simulations: Uniform Deployment of Nodes

**Figure 4:**
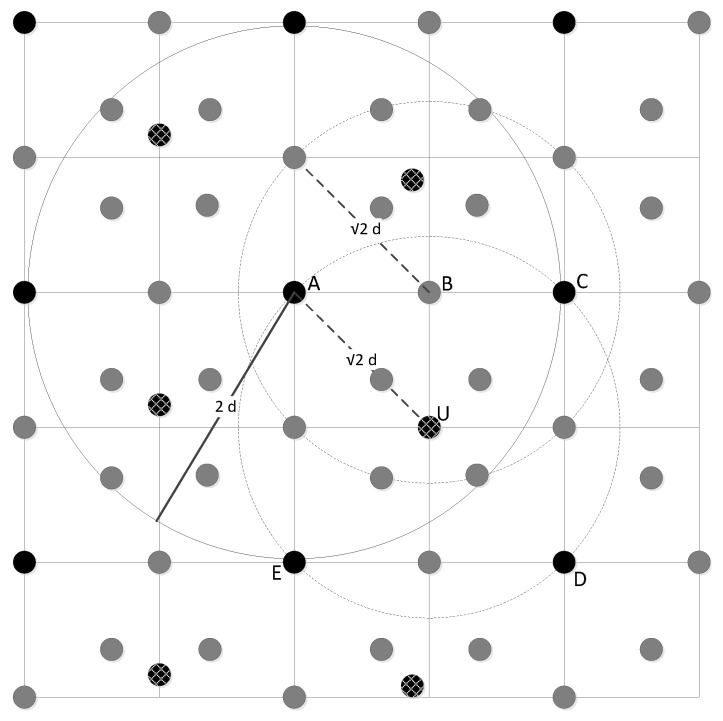
Uniform deployment.

In this first set of simulations, we assume that a set of sensor nodes is uniformly deployed. Figure 4 depicts this scenario, where solid black dots are representing the anchor (i.e., sensor nodes equipped with GPSs) nodes (e.g., *A*, *C*, *D*, *E*), and grey dots represent regular location-known sensors (e.g., *B*). The communication range of an anchor node is assumed to be 2d, where *d* equal to the distance between an anchor and any of its surrounding location-known sensor nodes. The communication range of a regular location-known sensor is $\sqrt{2}d$.

Any anchor node or regular sensor node may be a colluder. White-patterned-black dots represent location-unknown sensor nodes, e.g., sensor *U*. The communication range of a location-unknown sensor node is also $\sqrt{2}d$. We consider the worst case, in which the third category of colluders defined in Section 3 conduct the attacks. Under this scenario, node *U* (assumed to be a node that initially does not know its own position in the WSN) succeeds at detecting the attack of *B* if there are at least two more truth-tellers placed within the intersection of discs $d_{B}$ and $d_{U}$ (respectively centred at *B* and *U*) than the number of colluders.

**Figure 5:**
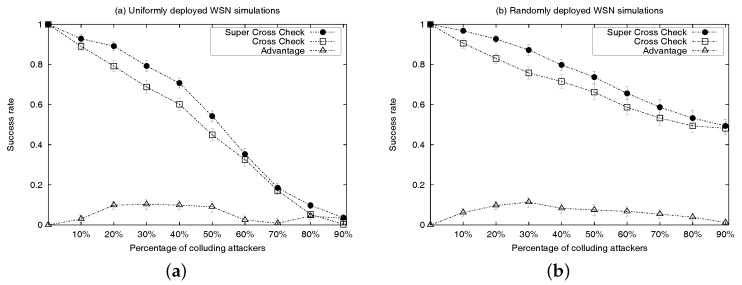
Comparative representation of the successful detection rate with regard to the super cross-check (SCC) algorithm and the cross-check (CC) algorithm. *Advantage* represents the difference between the success rate of the SCC and CC algorithm results. (**a**) Uniform deployment; (**b**) Random deployment. WSN: wireless sensor network.

**Figure 6:**
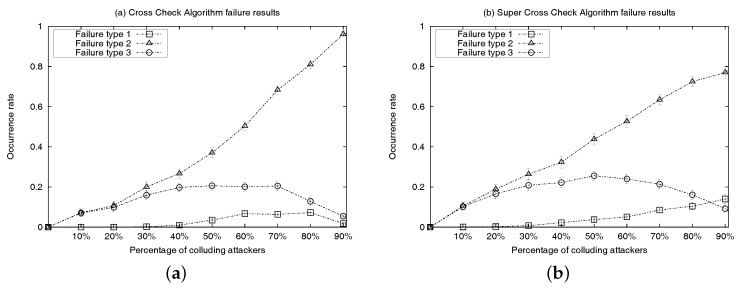
Comparison of colluder detection failures in the uniformly deployed WSN simulations. *Failure type 1* refers to a final CC list that is empty. *Failure type 2* refers to a non-empty final CC list containing, at least, one attacker. *Failure type 3* means a non-empty final CC list containing exactly one attacker. (**a**) CC algorithm; (**b**) super cross-check algorithm.

Simulation results are presented in Figure 5a and Figure 6. Each simulation represents the deployment, on a 12-cell grid, of 99 location-known nodes (20 sensor nodes are anchors and the remainder 79 are regular location-known nodes) and 12 location-unknown sensor nodes. Any location-known sensor can be a colluder. At every run, attackers are distributed randomly and uniformly within every cell of the grid (e.g., cells depicted as ACDE in Figure 4). Figure 5a shows the detection rate of location-unknown nodes that successfully detect colluders after executing algorithm CC or SCC, under the uniform sensor deployment scenario with incremental percentage of location-known sensors acting as colluders ranging from 10% to 90%. Results are measured using 100 runs per each percentage-of-attackers case and 95% confidence intervals. Success means that all colluders in the neighbourhood of a location-unknown node (executing either the CC or SCC algorithm) are successfully removed from the final list of truth-telling nodes. Figure 6 shows different types of failures when executing each algorithm under the uniform sensor deployment scenarios. Counted failures are cases in which either colluders are not properly removed from the final list of truth-telling nodes or when the final list ends being wrongly empty. Failures are due to the fact that the detection rate of both algorithms is not always 100% in our simulations. This is because in our simulations, we are not filtering out topologies in which the hypotheses of the algorithms are not met. In order to maximize the detection rate for SCC, the rate of category three colluders among all other colluder types shall be inversely proportional to the rate of colluders among all anchor nodes. Since attackers are distributed randomly and uniformly in every simulation run, a percentage increase of colluders also represents an increase of detection failure due to hypothesis violations (e.g., percentage of truth-telling nodes less than twice the percentage of colluders in the intersection of discs $d_{A}$ and $d_{B}$ centred at *A* and *B* in Figure 4). The *advantage* curve in Figure 5a represents the difference between the success rates of SCC and CC. It confirms that the detection success rate of SCC always outperforms that of CC. Looking at Figure 6, we can observe that the performance increase responds to the coverage of the colluding factor, so that more combinations of attackers are identified and removed from the final list of truth-telling nodes.

### 6.2. Second Set of Simulations: Random Deployment of Nodes

**Figure 7:**
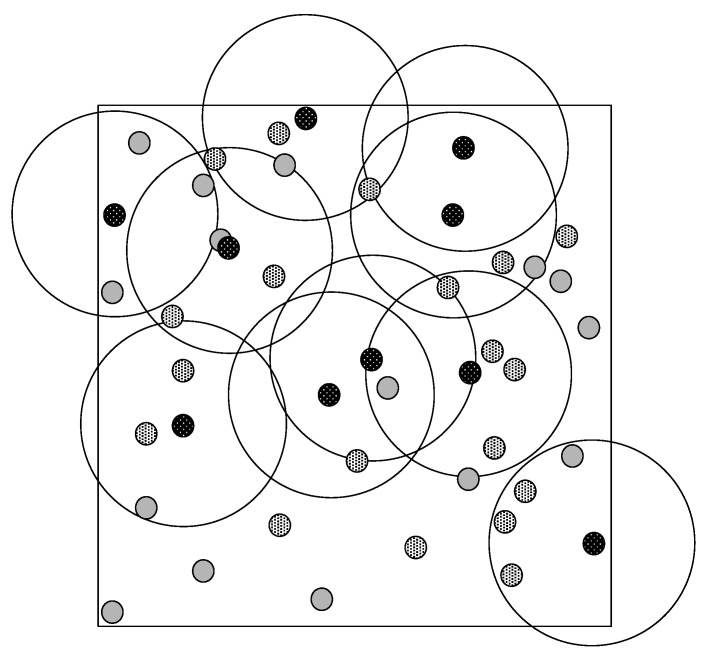
Random deployment.

In this section, we report numeric simulation in which all sensor nodes, both regular location-known and location-unknown nodes, are positioned at random. As in the uniform deployment case, any regular location-known sensor can be a colluder. Figure 7 depicts a sample random deployment of 44 sensor nodes, consisting of 34 location-known nodes and then nodes of type location-unknown. Of the 34 location-known nodes, 15 are acting as colluder nodes. Black-patterned white dots represent location-unknown nodes executing algorithm CC or SCC. White-patterned black dots represent regular location-known nodes acting as truth-telling sensors. Finally, grey dots represent colluder nodes. Solid black circles represent the communication range of the location-unknown sensor nodes.

Figure 5b contains the simulation results. Each simulation corresponds the deployment of eighty nodes of type non-colluders, and ten nodes of location-unknown type. An incremental number of additional location-known sensors act as colluders. The percentage of colluders is presented in proportion to the number of location-known sensors acting as truth-telling nodes (e.g., 10% of colluders means a WSN with eight location-known sensors acting as colluders in addition to the eighty nodes of type location-known acting as non-colluders; and ten regular nodes of type location-unknown). Results focus on the worst case, i.e., the case in which colluders are positioned randomly and uniformly within the WSN. Results are measured using 100 runs per percentage-of-attacker case and 95% confidence intervals. The SCC and CC curves in Figure 5b show the success rate of randomly positioned location-unknown nodes at detecting colluders after executing either algorithm. Success means that all neighbouring attackers of a location-unknown sensor node are successfully identified and removed from a final list of truth-telling nodes prior computation of the routing tables. The *advantage* curve in Figure 5b represents the difference between the success rate of SCC and CC. We can see from this curve that the detection success rate of SCC is superior to the detection success rate of CC under the random sensor deployment scenario. The very narrow 95% confidence intervals of the simulation results reflect the stability of the two algorithms in the random case.

### 6.3. Comparative Evaluation on Cost

**Figure 8:**
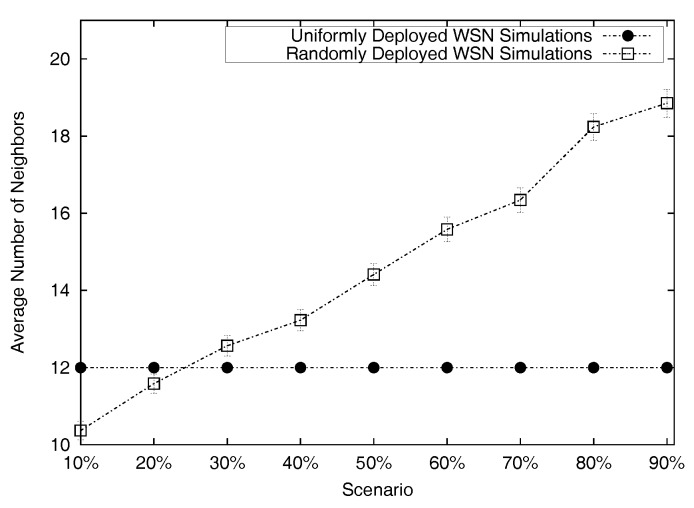
Average number of neighbours per location-unknown node and its 95% confidence intervals.

Figure 8 plots the average number of neighbours per location-unknown node, plus the 95% confidence intervals in each scenario. As expected, the number is always below 30 active neighbours In the uniform deployment simulations, the number of active neighbours per location-unknown nodes is always 12 neighbours This is because, in the uniform case, location-unknown nodes are always covered by four anchors and eight regular (non-anchor) location-known nodes. In the random case, there is no predetermined number of location-known nodes covering a location-unknown node. We recall that the topologies in the random deployment case consist of the random placement of exactly 80 non-colluders, 10 location-unknown sensors and an incremental number of additional location-known sensors acting as colluders. For random deployment, the curve in Figure 8 was computed by averaging the exact number of neighbours per location-unknown node in each of the 100 runs per simulation scenario (i.e., ten scenarios, from 10% to 90% colluders).

**Figure 9:**
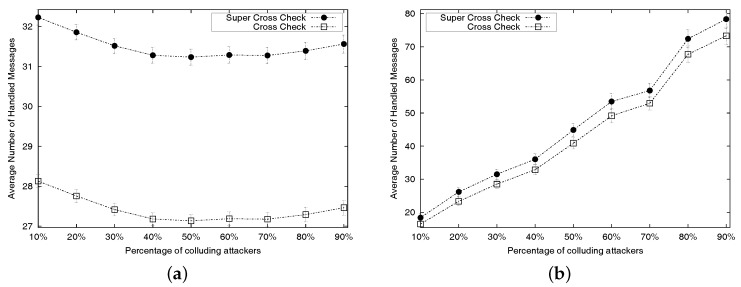
Average cost per algorithm and its 95% confidence intervals. (**a**) Uniform deployment; (**b**) Random deployment.

As a function of the number of active neighbours per location-unknown node, we compare the cost of executing algorithms SCC and CC. Figure 9 shows the average cost per algorithm, plus the 95% confidence intervals. The cost is determined by the amount of exchanges that a location-unknown node needs to handle. The description of Algorithms 1 and 2 shows that such cost mainly depends on the number of active neighbours; that is, the cost is bounded by the number of processed replies (cf. Lines 2 and 8 of Algorithm 1 for SCC; and Lines 2 and 7 of Algorithm 2 for CC). We can see that the number of processed replies in the SCC algorithm is always slightly higher than the equivalent number of processed replies in the CC algorithm. This leads to the conclusion that the cost of SCC is always higher than the cost of CC. The experimental results depicted in Figure 9 show that the increase is generally reasonable.

## 7. Conclusions

The deployment of a wireless sensor network (WSN) under hostile environments may lead to vulnerable scenarios. In particular, many distinct attacks corrupt the estimation of positions, which, in turn, can have a significant negative impact on the behaviour of software relying on such estimations. Consequently, much work has focused on how to correctly compute the latter in the presence of different kinds of attacks. However, how to deal with colluders has not received much attention. To remedy this, here we have focussed on three kinds of such attackers that target the localization process. Most importantly, these attackers are taken to be able to alter the information transmitted between the nodes of the WSN. We described at length a decentralized algorithm that established the coordinates of location-unknown nodes in the presence of such adversaries. Our solution also supports the detection of colluders within the communication range of this sensor. Through extensive simulations we validated two different environments: uniform deployments and random distribution of nodes. With regard to previous work, our new algorithm achieves a higher performance in terms of collusion detection, and at a reasonable cost increase.
